# Trends in NICE technology appraisals of non-small cell lung cancer drugs over the last decade

**DOI:** 10.1007/s10198-024-01711-0

**Published:** 2024-08-30

**Authors:** Lotte Westerink, Sharon Wolters, Guiling Zhou, Arjan Postma, Cornelis Boersma, Job Frank Martien van Boven, Maarten Jacobus Postma

**Affiliations:** 1 Department of Health Sciences, University of Groningen, University Medical Center Groningen, Hanzeplein 1, Groningen, 9713 The Netherlands; 2 AstraZeneca, Cambridge, UK; 3 Asc Academics B.V, Groningen, The Netherlands; 4 Unit of Pharmaco-Therapy, -Epidemiology and -Economics (PTEE), Department of Pharmacy, University of Groningen, Groningen, The Netherlands; 5 Health-Ecore B.V, Zeist, The Netherlands; 6 Department of Management Sciences, Open University, Heerlen, The Netherlands; 7 Department of Clinical Pharmacy & Pharmacology, University of Groningen, University Medical Center, Groningen Research Institute for Asthma and COPD (GRIAC), Groningen, The Netherlands; 8 Department of Economics, Econometrics & Finance, Faculty of Economics & Business, University of Groningen, Groningen, The Netherlands; 9 Center of Excellence in Higher Education for Pharmaceutical Care Innovation, Universitas Padjadjaran, Bandung, Indonesia

**Keywords:** NSCLC, HTA, Cost-effectiveness, Review, Technology appraisals, NICE, UK

## Abstract

**Objectives:**

The aim of this study is to analyse the trends in technology appraisals for non-small cell lung cancer (NSCLC) treatments performed by the National Institute for Health and Care Excellence (NICE) over the last ten years.

**Methods:**

A systematic search was conducted for single technology appraisals of NSCLC drugs in the online NICE database from 2012 to 2022. Search terms used were ‘non small cell lung cancer’, and ‘NSCLC’. Appraisals that were under development or terminated as well as multiple technology appraisals were considered out of scope.

**Results:**

In the 30 included appraisals for targeted therapies and immunotherapies within NSCLC, a total of 53 different comparators were included by NICE for 41 assorted indications or subgroups. Partitioned survival models were most frequently used, often including three health states and time horizons of up to 30 years. Throughout the decade the use of indirect comparisons was high and became more established and complex over time. Of all appraisals, 90% positively recommended the treatment for use in the UK.

**Conclusion:**

Technology appraisals became more complex over time due to the emergence of targeted therapies and immunotherapies, leading to multiple different indications, subpopulations and comparators that needed to be included in appraisals. Partitioned Survival Analysis (PartSA) models became the cornerstone within NSCLC, with time horizons up to 30 years and over time methods for indirect treatment comparisons became more established. The majority of the appraisals resulted in a positive recommendation for reimbursement.

**Supplementary Information:**

The online version contains supplementary material available at 10.1007/s10198-024-01711-0.

## Key points

• Lung cancer is the leading cause of cancer mortality, with about 80% of lung cancer patients diagnosed with non-small cell lung cancer (NSCLC). The objective of this study was to analyse the trends in National Institute for Health and Care Excellence (NICE) technology appraisals for NSCLC drugs in the United Kingdom over the last decade.

• Our study showed that Partitioned Survival Analysis (PartSA) models with the use of indirect treatment comparisons are currently the cornerstone within NSCLC, with included time horizons up to 30 years. Due to the emergence of targeted therapies and immunotherapies over the last decade, fast pace of updates in standard of care within NSCLC were seen. This has led to the required inclusion of multiple indications, subpopulations and comparators into the appraisals, resulting in an increased level of complexity.

• Technology appraisals in NSCLC will become even more intricate with an increased importance for more guidance on the elaboration of indirect comparisons, as new mutations and expressions are still being identified and innovative therapies are emerging, such as treatments for the mutations or expressions BRAF, HER2, HER3, KRAS, MET, and pan-tumour molecular targets.

**Supplementary Information:**

The online version contains supplementary material available at 10.1007/s10198-024-01711-0.

## Introduction and objective

Lung cancer is the leading cause of cancer mortality, with about 80% of lung cancer patients diagnosed with non-small cell lung cancer (NSCLC) [1, 2]. Next to a high mortality rate, lung cancer is associated with high morbidity and healthcare costs [1]. Over the last decade, systemic anticancer therapy for advanced or metastatic NSCLC evolved to more personalized therapy based on tumour histology and oncogene biomarker status. In this period, new NSCLC treatments emerged with focusing more on specific mutations or expressions like anaplastic lymphoma kinase (ALK), epidermal growth factor receptor (EGFR), c-ros oncogene 1 (ROS1) and programmed death-(ligand) 1 (PD-(L)1) expression. These emerging innovative therapies are leading to better patient outcomes such as improved overall survival, but also come with a significant burden on healthcare budgets [3]. 

Globally, governments need to allocate healthcare interventions within limited healthcare budgets. Economic evaluations are increasingly being conducted to estimate a value-based cost-effective (negotiated) price for treatments. In the UK, the cost-effectiveness of interventions is of crucial importance in the decision-making process by the National Health Service (NHS), which is informed by guidance and recommendations of the National Institute for Health and Care Excellence (NICE) [4]. NICE is one of the global leaders in performing health technology appraisals. Since July 2016, all new cancer drugs are considered for recommendation into the Cancer Drugs Fund (CDF) when there is significant remaining clinical uncertainty as well as the related uncertainty about the cost-effectiveness [5]. After the period in the CDF, the treatment will be re-evaluated for recommendation in the NHS. To evaluate an intervention with regards to the cost-effectiveness, NICE considers it most appropriate to use an acceptable range for the incremental cost-effectiveness ratio (ICER) as a willingness-to-pay (WTP) threshold. Judgement on the WTP threshold above £20,000 per quality-adjusted life year (QALY) considers a combination of the following factors: degree of certainty around the ICER, the way the change in health-related quality of life (HRQoL) is captured, the innovative nature of the technology, meeting end-of-life criteria, and non-health related aspects [6]. When a treatment is considered life-extending at the end of life, usually the NICE applies a WTP threshold of £50,000 per QALY. Two key criteria need to be met for this consideration: (1) The treatment is indicated for patients with a short life expectancy (< 24 months), (2) There is sufficient evidence to indicate that the treatment’s prospect has a mean value of three months additional extension to life in comparison to the current NHS treatment [6]. The assumption is that when treatments for new mutations become available, NICE will first consider a WTP threshold of £50,000 per QALY. When more treatments for the same mutation will come available to the market, the WTP threshold for first line treatment will decrease to £20,000–30,000 per QALY and for second line treatment this will then become £50,000 per QALY.

Over the last decade various reviews of economic evaluations for interventions in oncology and in NSCLC have been published. Multiple studies analysed how survival was modelled for cancer treatments in NICE appraisals [7–10]. These studies were focused on appraisals performed by NICE in oncology although these were not focused on treatments within NSCLC. When considering reviews that focused on NSCLC, these reviews did not consider appraisals performed by NICE, but analysed cost-effectiveness of targeted mutations, such as EGFR and ALK, [11, 12] or immune checkpoint inhibitors within NSCLC [13, 14]. Another study focused on economic evaluations in second and later lines of therapy in NSCLC, but this study only included first generation tyrosine kinase inhibitors (TKIs) and pemetrexed, which is limited in the context of all emerging medicine developments in the field of NSCLC [15]. Also, this study reported lifetime time horizons that only concerned two to three years, which was considered similar as the terminal nature of the disease and the high mortality rates [15]. Current technology appraisals in the UK usually consider a time horizon between 10 and 30 years depending on mutation or expression, which is also in line with the major clinical developments in the treatment of NSCLC seen over the last ten years.

It is to be expected that due to the above-mentioned dynamic landscape, the economic evaluations of treatments of NSCLC changed over the years. Analysing NICE technology appraisals of NSCLC-treatments over the last ten years potentially uncovers relevant trends. Therefore, the objective of this study is to analyse the trends in NICE technology appraisals for NSCLC drugs in the last decade. This study focuses on the indications within NSCLC, model specifics and outcomes, indirect treatment comparisons, and recommendations. The outcomes of our study potentially inform the development and market access process of new oncology therapies with regards to what steps need to be considered both from a clinical outcomes research perspective as well as a health economics perspective.

## Methods

### Study design

The website of NICE contains a transparent database for all technology appraisals and other guidance. This review focused on Single Technology Appraisals (STAs), designed to appraise an intervention for a single indication, for therapies of NSCLC over the last decade.

A systematic search was conducted on January 27, 2022, for published STAs in the online database of NICE in the UK (nice.org.uk) between the period: 1 January 2012 and 1 January 2022. The search terms used concerned ‘non small cell lung cancer’, and ‘NSCLC’. The applied filters concerned Source – ‘NICE’, and Evidence type – ‘Guidance and Policy’ and ‘Prescribing and Technical information’. Terminated appraisals, appraisals in development and Multiple Technology Appraisals (MTAs) were out of scope of this study. All appraisals were screened independently by LW and SW. Any disagreements were resolved by consensus.

### Data extraction

Data from each technology appraisal was extracted from the most recent committee papers and committee slides from NICE. When specific data was not found in the most recent guidance due to redacted text and data (black boxes) or references to earlier appraisal rounds, additional data was extracted from committee papers of former appraisal rounds when available. Data from budget impact analyses, which are a standard part of the technology appraisal, were predominantly not publicly available and were therefore out of scope.

### Data items

A data extraction form was created to corroborate the needed information to meet our study objective. Extracted data included disease area and indication (generic name, mutation/expression, line of treatment), overview of technology appraisal details (publication date, recommendations, uptake in CDF), economic model characteristics (model structure, time horizon, cycle length), cost-effectiveness outcomes and WTP thresholds, uncertainty analyses, patient reported outcome measures, comparators, and indirect treatment comparisons. See appendix A for further details of the extraction form.

### Outcomes

This review aimed to identify timely trends due to the clinical developments in NSCLC with regards to overall trends in the treatment landscape, recommendations by NICE, cost-effectiveness models used and the inclusion of relevant comparators for (in)direct treatment comparisons, and possible trends in patient reported outcomes measures.

## Results

### Screening process

The initial search yielded 119 records, of which 55 were excluded due to the following reasons: other indication focus (*N* = 25), appraising a non-drug medical technology (*N* = 7), concerned disease management guidance (*N* = 13), information update (*N* = 1), concerned a multiple technology appraisal (*N* = 1), and appraisal was terminated (*N* = 8). Also, records were excluded as these were updated and replaced by other TA numbers, and deduplication of the resulting 60 records excluded another 30 records. Finally, this resulted in a total of 30 technology appraisals for treatments in NSCLC that were included for the data extraction.

### Data extraction

The included appraisals concerned eleven indications including PD-L1 expression, eight for ALK mutations, six for EGFR mutations, two for the ROS1 oncogene, one for NSCLC of adenocarcinoma histology (nintedanib), one for previously treated NSCLC (ramucirumab), and one for maintenance treatment for NSCLC (pemetrexed).

Among these technology appraisals, 17 concerned first line indications. One of the 17 appraisals concerned an indication for first and later lines (crizotinib TA529) and another appraisal (atezolizumab TA584) included both first line treatment for PD-L1 expression of 0–49% and as well as later line when targeted therapy for EGFR-positive or ALK-positive NSCLC failed. In addition, 12 appraisals concerned second line indications of which two appraisals indicated second and later lines (pembrolizumab TA428, and lorlatinib TA628). Lastly, one technology appraisal for pemetrexed concerned maintenance treatment.

**Fig. 1:**
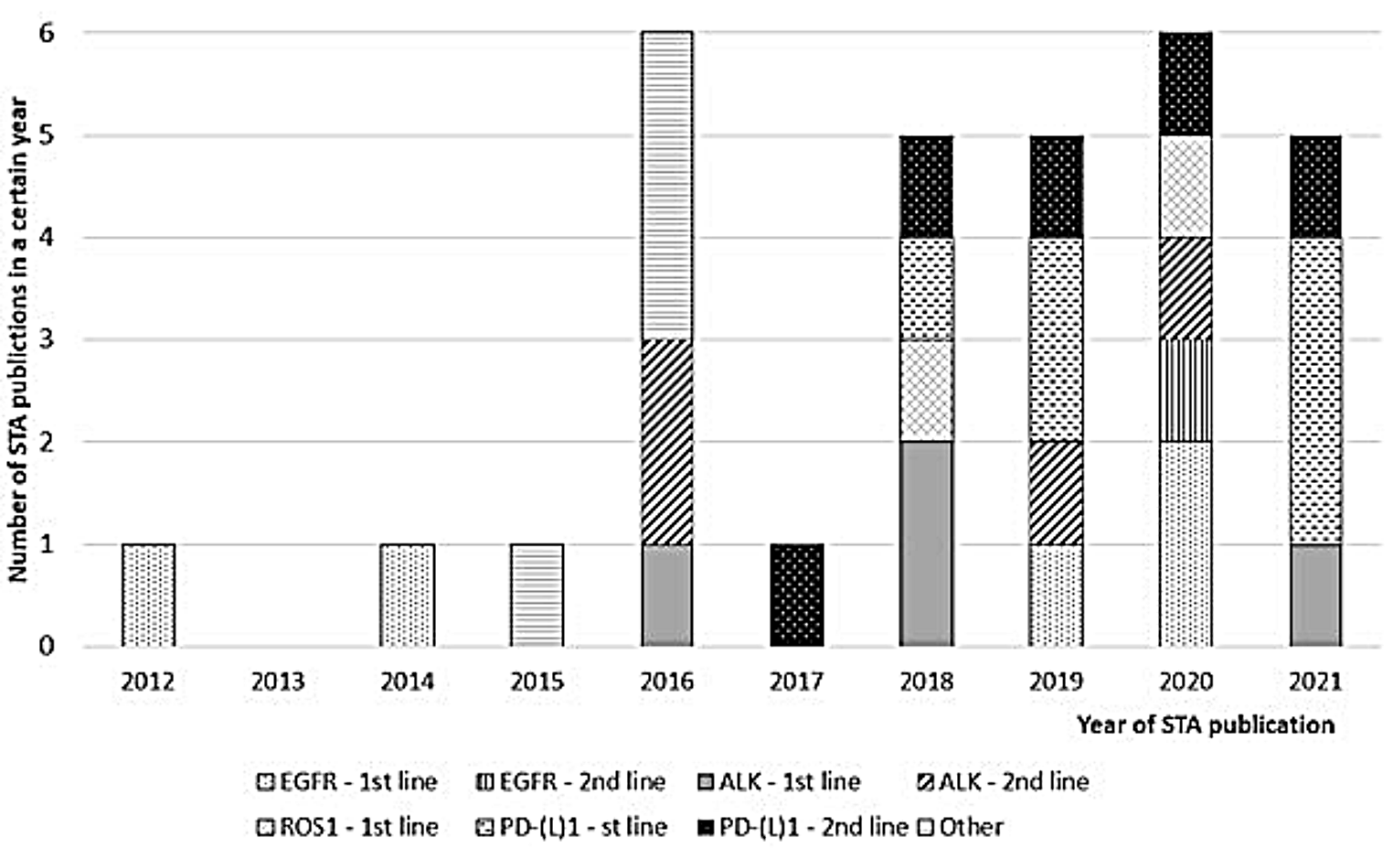
Overview of number of STA publications per year differentiated per first- and second line indications. *Abbreviations* ALK: Anaplastic lymphoma kinase, EGFR: Epidermal growth factor receptor, PD-(L)1: Programmed Death-Ligand 1, ROS1: c-ros oncogene, STA: Single Technology Appraisal

In Fig. 1 and in more detail in Table 1 an overview is provided of the extracted NICE technology appraisals 2012–2022 in chronological order of publication year. Trends will be further discussed per mutation or expression with a focus on mutations EGFR, ALK and ROS1, and on PD-(L)1 expression.

### Trends in NSCLC treatments landscape in technology appraisals

#### EGFR mutation positive NSCLC

EGFR TKIs were one of the first molecular targeted therapies in NSCLC and replaced platinum-based combination chemotherapy as first line therapy for patients with EGFR-positive NSCLC. These treatments emerged rapidly in one decade with three generations of TKIs succeeding one another. Technology appraisals of the EGFR TKIs are mainly indicated for first line indications (see Fig. 1). With the two appraisals of the third generation TKI osimertinib in 2020, a new trend for the EGFR TKIs seems to be introduced in which first the second line indication will be appraised, and the first line appraisal will follow consecutively.

#### ALK- and ROS1 positive mutations in NSCLC

Improved understanding of oncogenes like ALK led the TKI crizotinib to be the first treatment appraised and recommended drug for NSCLC with ALK gene rearrangements in the UK. After the first appraisal of crizotinib for first line treatment of ALK-positive NSCLC, four other TKIs (ceritinib, alectinib, brigatinib and lorlatinib) were also appraised. These last four treatments were first appraised for the second line indication, followed by the first line indications (see Fig. 1). Crizotinib also received a positive recommendation for second line treatment.

In 2018, the indication of crizotinib was expanded and included also first- and later line treatment for NSCLC patients with a ROS1 rearrangement. Together with entrectinib, which received a positive recommendation in 2020, these two treatments were the only two drugs recommended for ROS1 positive NSCLC in the UK.

#### NSCLC with PD-L1 expression

From 2015 onwards, the first technology appraisals for immunotherapies in NSCLC were assessed. Currently, only four compounds (pembrolizumab, nivolumab, atezolizumab and durvalumab) were appraised for a total of eleven indications. These indications can vary with regards to squamous or non-squamous NSCLC, tumour proportion score (TPS) (PD-L1 expression of ≥ 1%; 0–49%; or ≥ 50%), with or without EGFR/ALK mutations, first- or second line, mono- or combination therapy. What stands out is that these pan-tumour drugs are also amenable to targeted therapy for mutations like EGFR and ALK. Overall, second line indications were first appraised, and first line indications followed from 2018 onwards (see Fig. 1).

In the first years of assessing PD-(L)1 inhibitors, more than one appraisal round was needed for an NHS recommendation. It appeared that both companies and NICE needed to find a new way to appraise this immunotherapy category.

### Overall trend in treatment landscape

Overall, when a new targeted mutation is identified, it seems that often the first treatment to be appraised by NICE concerns a first line indication when there is a high medical unmet need (see Fig. 1). The expectation for subsequent emerging treatments for the same mutation is that these will first be appraised for the second line indication and that the appraisal of the first line indication will follow consecutively. For new treatments like immunotherapies that focus on a certain expression, there is a high possibility that second line indications will be appraised first as there are already other treatment options available and used when the treatment emerges.

### Overview recommendations NICE

**Fig. 2:**
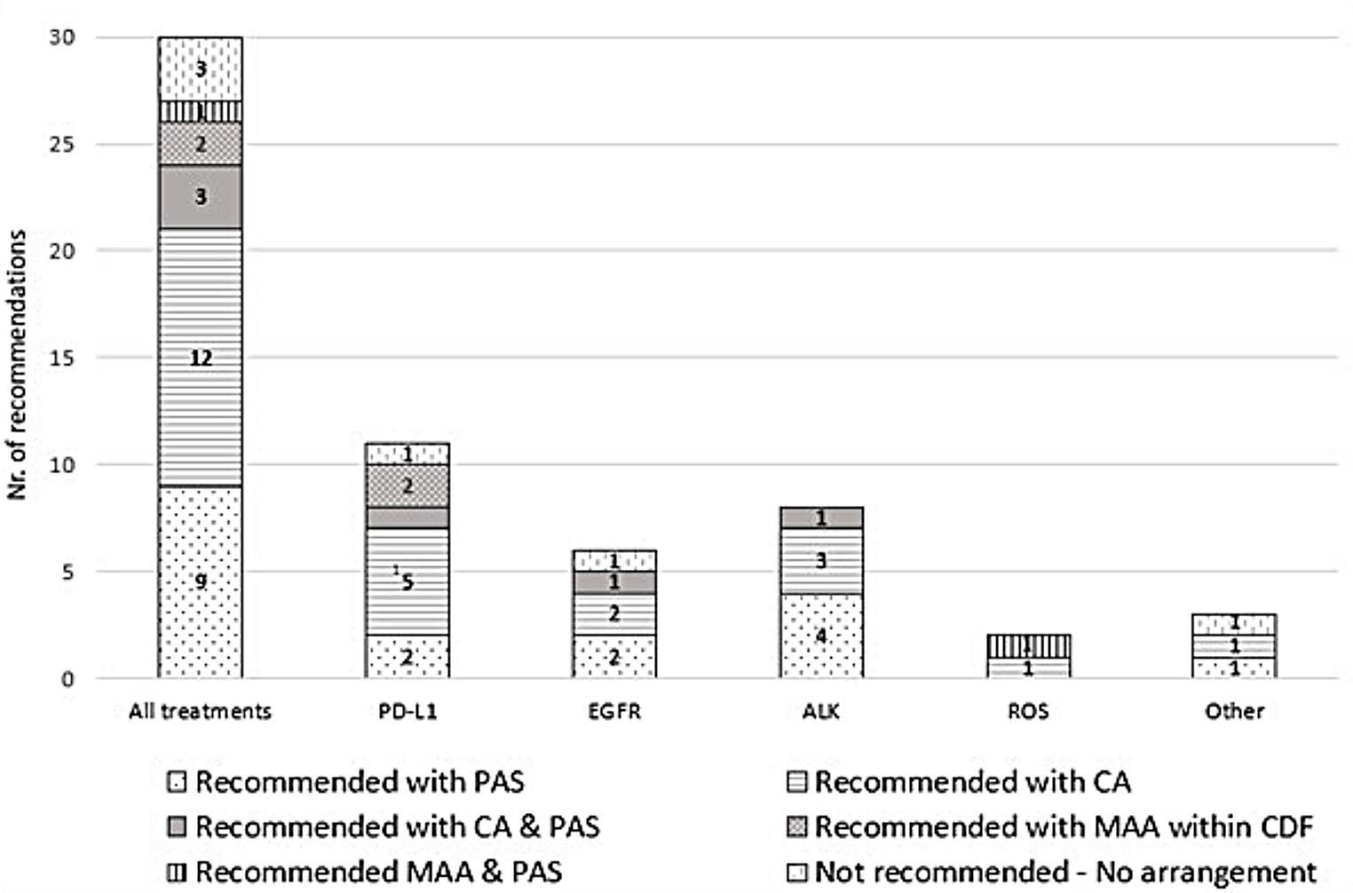
Overview of recommendations following NICE technology appraisals of NSCLC treatments for mutations EGFR, ALK and ROS1, for expression PD-L1, and Other. *Abbreviations* ALK: Anaplastic lymphoma kinase, CA: Commercial Agreement, CDF: Cancer Drug Fund, EGFR: Epidermal growth factor receptor, MAA: Managed Access Agreement, PAS: Patient Access Agreement, PD-L1: Programmed death-ligand 1, ROS1: c-ros oncogene

Figure 2 provides an overview of NICE recommendations for NSCLC’s technology appraisals; currently, 24 out of 30 appraisals were recommended for NHS (of which four appraisals were previously recommended in CDF), three were recommended for CDF (TA529, TA578, and TA600), and three were not recommended (TA403, TA411, and TA724). All recommendations for NHS and for CDF included market access agreements, varying from commercial agreement (CA) or commercial access agreement (CAA), patient access scheme (PAS), to managed access agreement (MAA). No trend could be found in these commercial arrangements with regards to indications, the treatment’s mechanisms of action, or over time. The content of the commercial arrangements was not public, although for 15 technology appraisals it was mentioned that the CA or PAS concerned a simple price discount.

The above-mentioned three treatments that were not recommended concerned indications for second line ramucirumab (TA403), and both first line necitumumab (TA411) and nivolumab (724). Both treatments ramucirumab and necitumumab showed clinical effectiveness but the most plausible ICERs were well over the range that would normally be considered a cost-effective use of NHS resources. The clinical efficacy for nivolumab in combination with ipilimumab and 2 cycles of chemotherapy has only been compared indirectly with other treatments. These indirect comparisons results were uncertain, and the cost-effectiveness estimates were uncertain and higher than what is considered acceptable. For this treatment it was decided that there is no additional data that could be collected through CDF or from clinical trials to resolve this uncertainty.

### Cost-effectiveness models

**Fig. 3:**
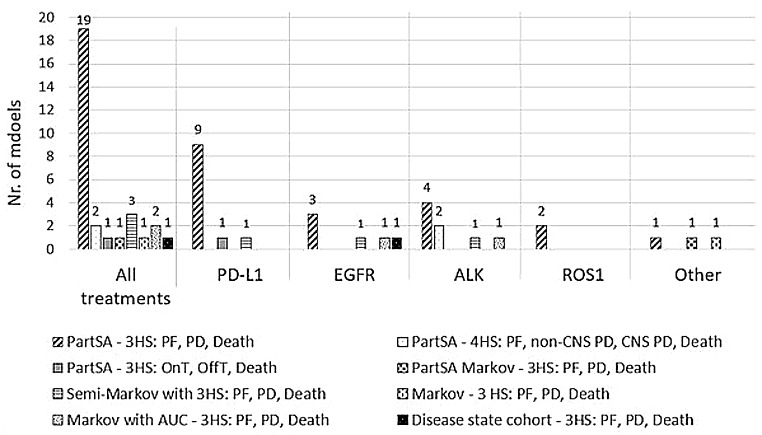
Overview of economic models used in technology appraisals, categorized as all treatments and per receptor groups: PD-(L)1, EGFR, ALK, ROS1, Other. *Abbreviations* ALK: Anaplastic lymphoma kinase, AUC: Area under the curve, CNS: Central Nerve System, EGFR: Epidermal growth factor receptor, HS: Health State, OffT: Off Treatment, OnT: On treatment, PD: Progressed Disease, PD-L1: Programmed death-ligand 1, PF: Progression Free, PartSA: Partitioned Survival Analysis, ROS1: c-ros oncogene 1

The cost-effectiveness models that were performed until 2016 were mainly (semi-)Markov models or Markov models with ‘area under the curve (AUC) partitioned survival analysis (PartSA) technique’. Increasingly, PartSA models were increasingly used and became a frequently used modelling approach within NSCLC. In Fig. 3, an overview of economic models is presented categorized per treatments and mutation/ expression: PD-L1, EGFR, ALK, ROS1. Out of the 30 technology appraisals, 27 appraisals included three health transitions or states concerning progression free (PF) or pre-progression, progressed disease (PD) or post progression, and death (Fig. 3). A trend that was seen for three technology appraisals of PD-(L)1 inhibitors (TA428, TA584, and TA683) that included these three health transitions, concerned the inclusion of utility values using the proximity to death approach. This implies that more than one death-related time category per health state was included into the model, for example: <30 days or > 30 days to death, or even more time categories like ≤ 5 weeks before death, 5–11 weeks before death, 15–30 weeks before death, > 30 weeks before death. Atezolizumab was appraised for three indications. Notably, the first out of three appraisals used an alternative model by including the following three health states: ‘on treatment’, ‘off treatment’ and ‘death’. Two appraisals included four health transitions (progression free, non-central nervous system (CNS) progression, CNS progression and death) instead of three, these concerned PartSA models for both alectinib and brigatinib for the treatment of first line ALK-positive NSCLC (TA536 and TA670). In this way non-CNS disease and CNS disease could be captured separately.

**Fig. 4:**
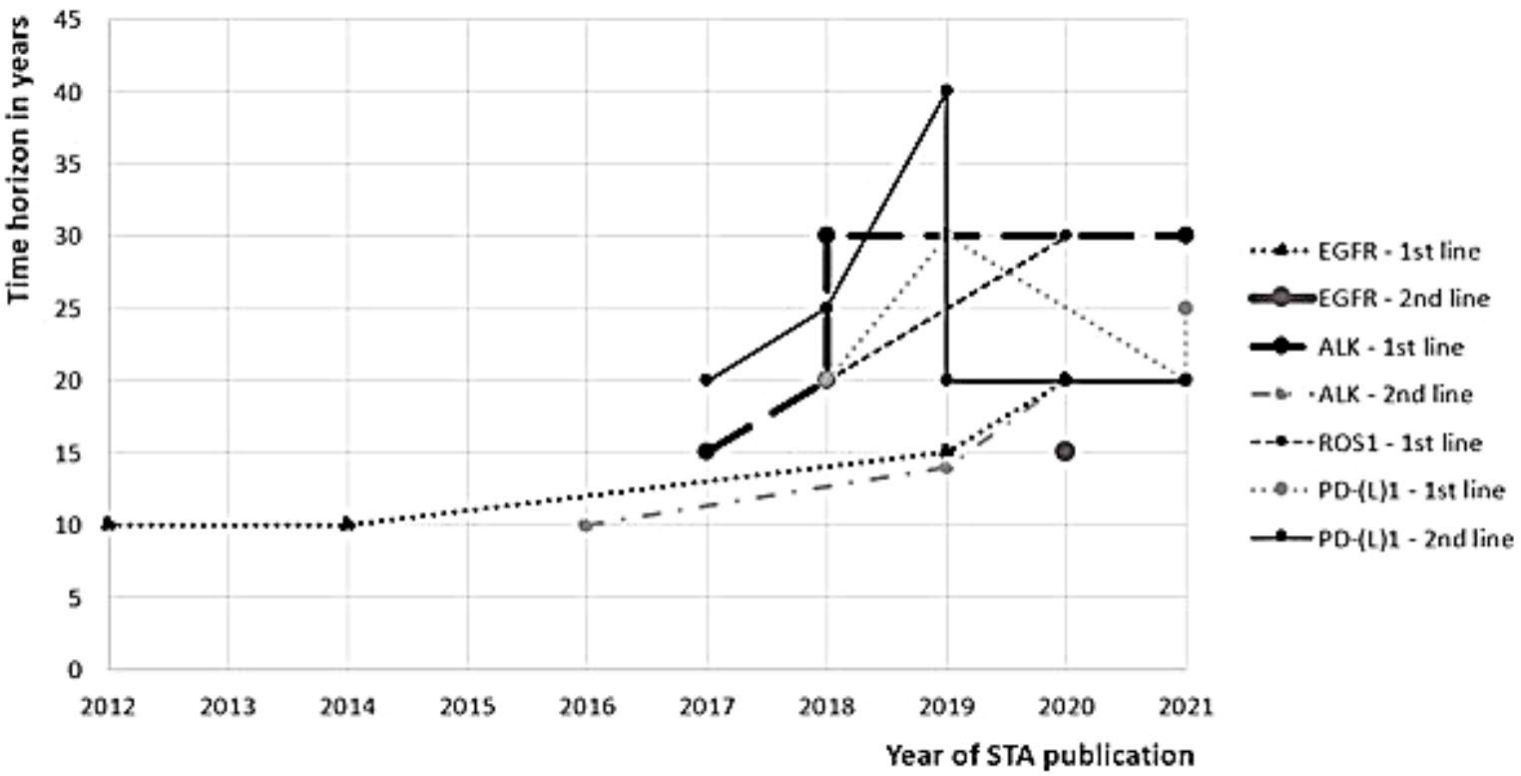
Overview of time horizons applied in cost-effectiveness models categorized per line of therapy and receptor groups: EGFR, ALK, ROS1, PD-(L)1. *Abbreviations* ALK: Anaplastic lymphoma kinase, EGFR: Epidermal growth factor receptor, PD-L1: Programmed death-ligand 1, ROS1: c-ros oncogene 1

Cycle lengths for PD-(L)1 inhibitors concerned predominantly seven days (10 out of 11 models), in contrast to models of NSCLC with positive EGFR, ALK or ROS1 mutations. In these models, the majority (13 out of 17 models) applied a cycle length of 28 to 30 days, and the other four technology appraisals applied a cycle length of 7 days. The approach for cycle length seemed to be unrelated to the time horizon. A lifetime horizon was frequently considered with different time horizons often applied as these suffice for a lifetime view in an often-fatal disease (See Table 1; Fig. 4). Over the last decade, the time horizon for economic models for EGFR positive NSCLC increased from 10 years to 20 years for the last technology appraisal concerning first line treatment with osimertinib.

**Table 1:** Chronological overview of NICE Technology appraisals 2012–2022 and model structures. *Abbreviations* ALK: Anaplastic lymphoma kinase, AUC: Area under the curve, BSC: Best supportive care, CNS: Central nervous system, D: Death, EGFR: Epidermal growth factor receptor, NSCLC: Non-small cell lung cancer, OnT: On treatment, OffT: Off treatment, PD: Progressed disease or post-progression, PD-L1: Programmed death-ligand 1, PEM: Pemetrexed, PLAT: Platinum chemotherapy, PF: Progression free or stated as pre-progression, PartSA: Partitioned survival analysis, QALY: Quality-adjusted Life Year, ROS1: C-ros oncogene 1, SA: sensitivity analysis, TA: Technology appraisal, TPS: tumour proportion score

TA	Publication year	Generic name (+ combination therapy)	Indication focused on mutation/ expression	Line of treatment	Model	Model health states (& transitions)	Time horizon	Cycle length (days)	Reference
258	2012	Erlotinib	EGFR	1st	Semi-Markov	3: PF, PD, D	10	30	[16]
310	2014	Afatinib	EGFR	1st	Disease state cohort model	3: PF, PD, D	10	30	[17]
347	2015	Nintedanib (+ docetaxel)	Adenocarcinoma	2nd	PartSA Markov	3: PF, PD, D	15	21	[18]
395	2016	Ceritinib	ALK	2nd	Markov, with ‘AUC PartSA’ technique	3: PF, PD, D	10	30	[19]
402	2016	PEM maintenance treatment	Non-squamous	Maintenance treatment	Markov	3: PF, PD, D	Lifetime	21	[20]
403	2016	Ramucirumab (+ docetaxel)	NSCLC	2nd	PartSA	3: PF, PD, D	15	21	[21]
406	2016	Crizotinib	ALK	1st	Semi-Markov, with ‘AUC PartSA’ technique	3: PF, PD, D	15	30	[22]
411	2016	Necitumumab (+ induction (gemcitabine + cisplatin), maintenance (necitumumab)	Squamous with EGFR	1st	Markov, with ‘AUC PartSA’ technique	3: PF, PD, D	15	7	[23]
422	2016	Crizotinib	ALK	2nd	PartSA	3: PF, PD, D	10	30	[24]
428	2017	Pembrolizumab	PD-L1, with/ without EGFR or ALK	2nd & later	PartSA	3: PF, PD, D	20	7	[25]
500	2018	Ceritinib	ALK	1st	PartSA	3: PF, PD, D	20	30	[26]
520	2018	Atezolizumab	NSCLC	2nd	PartSA	3: OnT, OffT, D	25	7	[27]
531	2018	Pembrolizumab	PD-L1 ≥ 50%, no EGFR or ALK	1st	PartSA	3: PF, PD, D	20	7	[28]
529	2018	Crizotinib	ROS1	1st & later	PartSA	3: PF, PD, D	20	30	[29]
536	2018	Alectinib	ALK	1st	PartSA	4: PF, non-CNS-PD, CNS-PD, D	30	7	[30]
571	2019	Brigatinib	ALK	2nd	PartSA - ‘AUC’	3: PF, PD, D	14	28	[31]
578	2019	Durvalumab	PD-L1 ≥ 1%	2nd	Semi-Markov (State transition)	3: PF, PD, D	40	14–28**	[32]
584	2019	Atezolizumab (+ bevacizumab + carboplatin + paclitaxel)	PD-L1 (< 50%), EGFR, ALK	A. 1st when PD-L1 0–49% B. Later line when EGFR/ALK therapy failed.	PartSA	3: PF, PD, D	20	7	[33]
595	2019	Dacominitib	EGFR	1st	PartSA	3: PF, PD, D	15	28	[34]
600	2019	Pembrolizumab (+ carboplatin + paclitaxel)	Squamous	1st	PartSA - ‘AUC’	3: PF, PD, D	30	7	[35]
628	2020	Lorlatinib	ALK	2nd & later	PartSA	3: PF, PD, D	20	30	[36]
643	2020	Entrectinib	ROS1	1st	PartSA, cohort based	3: PF, PD, D	30	30	[37]
653	2020	Osimertinib	EGFR T790M	2nd	PartSA	3: PF, PD, D	15	7	[38]
654	2020	Osimertinib	EGFR	1st	PartSA	3: PF, PD, D	20	30	[39]
655	2020	Nivolumab	Squamous	2nd	PartSA	3: PF, PD, D	20	7	[40]
670	2021	Brigatinib	ALK	1st	PartSA	4: PF, non-CNS PD, CNS-PD, D	30	28	[41]
683	2021	Pembrolizumab (+ PEM + PLAT)	Non-squamous, no EGFR or ALK	1st	PartSA	3: PF, PD, D	20	7	[42]
705	2021	Atezolizumab	PD-L1 ≥ 50% or 10% of tumour-infiltrating immune cells, no EGFR or ALK	1st	PartSA	3: PF, PD, D	20	7	[43]
713	2021	Nivolumab	Non-squamous PD-L1 pos.	2nd	PartSA	3: PF, PD, D	20	7	[44]
724	2021	Nivolumab (+ ipilimumab + PDC)	NSCLC, no EGFR or ALK	1st	PartSA	3: PF, PD, D	25	7	[45]

For NSCLC with ALK mutations, the time horizon also started at 10 years and increased to 20 years or higher for the majority of appraisals. Only the two models with four health transitions for first line treatment ALK-positive NSCLC included both a 30-year time horizon. Most of the technology appraisals for PD-(L)1 inhibitors included a time horizon of 20 years, and time horizons of 30 and 40 years were included once, respectively.

There is a tendency for first line treatments to include longer time horizons of 20 to 30 years. In second line treatments, time horizons of 15 years and 20 years were applied most often. Overall, the time horizon included in models increased to 20 years or higher over the years.

The applied WTP threshold in the technology appraisals that focused on first line treatment of EGFR-, ALK- or ROS1-positive NSCLC was £20,000 - £30,000 per QALY overall (Table 1, Appendix B). Only the first treatment available for first line treatment for a specific mutation did meet the NICE end-of-life criteria that led to a WTP threshold of £50,000 per QALY. This concerned crizotinib for first line treatment of ALK positive NSCLC (TA406) and entrectinib for first line treatment of ROS1 positive NSCLC (TA643). All appraisals for targeted second line treatments of EGFR, ALK and ROS1 positive NSCLC met the end-of-life criteria, leading to a WTP threshold of £50,000 per QALY. When looking at the technology appraisals for the PD-(L)1 inhibitors, thresholds showed to be £50,000 per QALY when end-of-life criteria were met, this concerned all first-time appraisals for a certain indication. For PD-(L)1 inhibitors, when another treatment was appraised for the same indication the WTP threshold was £20,000–30,000 per QALY, this not only concerned indications for first but also for second and later lines. Which is not in line with the WTP thresholds for targeted second line treatments like EGFR-, ALK-, and ROS1 inhibitors. In three of these 11 appraisals for PD-(L)1 inhibitors (TA520, TA683, and TA724) the evaluation of end-of-life criteria was performed in two or three subpopulations, leading to separate WTP thresholds per subpopulation.

In short, treatments that were appraised for one targeted population in particular like EGFR-, ALK- or ROS1-positive NSCLC, had one clear WTP threshold. Treatments, like PD-(L)1 inhibitors, that were appraised for an indication that covered multiple subpopulations, the WTP threshold was set per subpopulation depending on meeting the end-of-life criteria.

The results of the ICER were often presented (20 out of 30 appraisals) without stating whether the presented ICER included a confidential discount or not (see Table 1 in Appendix B). Within this group, there were eleven appraisals that presented an ICER that was above the WTP threshold. Also, six of these appraisals received a positive recommendation, two appraisals received a positive recommendation for CDF (TA529 and TA578), and three appraisals received a negative recommendation (TA403, TA411 and TA724 as discussed previously). In the other 10 out of the 30 appraisals, one appraisal presented publicly both the ICER with and without a patient access agreement, one appraisal stated that the list price was included, and the other eight appraisals were only presented with a discount commercial in confidence. Ergo, with the confidentiality involved, it is challenging to describe any further trends with regards to the ICER results.

Although performed for all, data on probabilistic sensitivity analysis as well as scenario analyses were not publicly available for two appraisals (no probabilistic sensitivity analysis available for TA310, and no scenario analyses available for TA258). It is assumed that all appraisals performed uncertainty analyses given data were publicly available on either one or two analyses (univariate, probabilistic or scenario) for the appraisals with unavailable data.

### Comparators and indirect comparisons

While the treatment landscape in NSCLC evolved from chemotherapy to targeted therapies and immunotherapy, this was reflected in comparators and subgroups considered in the technology appraisals. Head-to-head trials were often not available for all comparators and subgroups that needed to be included in the technology appraisals. In the 30 appraisals a total of 53 different comparators were included by NICE (Table 2) for 41 assorted indications or subgroups. In only 24 of these cases, comparators were included in head-to-head trials. Indirect comparisons were performed in 22 out of the 30 included appraisals (Table 1 in Appendix C). The methods mixed treatment comparisons (MTC) and indirect analysis were only observed from 2012 to 2016. From 2016 onwards, two new methods were increasingly applied, these concerned network meta-analyses (NMAs) and matching-adjusted indirect comparisons (MAICs). Three indirect treatment comparisons mentioned whether these either unadjusted or adjusted. One ITC and three NMAs concerned fractional polynomials which provided flexible parametric modelling for analysing time-to-event data.

**Table 2:** Chronological overview of comparators selected by NICE that needed to be included in the economic evaluations in the technology appraisals. *Abbreviations* ABCP: atezolizumab + bevacizumab + carboplatin + paclitaxel, ALK: anaplastic lymphoma kinase, BSC: best supportive care, EGFR: epidermal growth factor receptor, NSCLC: non-small cell lung cancer, Neg.: negative, PD-L1: programmed death-ligand 1, PDC: platinum-doublet chemotherapy, PEM: Pemetrexed, PLAT: platinum chemotherapy, Pos.: positive, ROS1: C-ros oncogene 1, SOC: Standard of care TA: technology appraisal, TKI: tyrosine kinase inhibitors, TPS: tumour proportion score

TA	Publication year	Generic name (+ combination therapy)	Indication focused on mutation/ expression	Line of treatment	Sub indications or subgroups for NICE comparators	NICE comparators	Same as comparator in RCT (Y/*N*)	RCT name	RCT comparator
258	2012	Erlotinib	EGFR	1st	1 Indication	Gefitinib	N	EURTAC	PDC
310	2014	Afatinib	EGFR	1st	1 Indication	Gefitinib	N	Lux-Lung 3	PEM + PLAT
					1 Indication	Erlotinib	N	Lux-Lung 6	PEM + PLAT
347	2015	Nintedanib (+ docetaxel)	Adenocarcinoma	2nd	1 Indication	Docetaxel	Y	LUME-Lung 1	Placebo + docetaxel
395	2016	Ceritinib	ALK	2nd	1 Indication	BSC	N	ASCEND 1	Single arm trial
					1 Indication	BSC	N	ASCEND 2	Single arm trial
403	2016	Ramucirumab (+ docetaxel)	NSCLC	2nd	Full population	Docetaxel	Y	REVEL	Placebo + docetaxel
					Subgroup squamous NSCLC	Docetaxel	Y	REVEL	Placebo + docetaxel
					Subgroup non-squamous NSCLC	Docetaxel	Y	REVEL	Placebo + docetaxel
					Subgroup non-squamous NSCLC	Nintedanib + docetaxel	N	REVEL	Placebo + docetaxel
402	2016	Pemetrexed maintenance treatment	Non-squamous	Maintenance treatment	1 Indication	BSC	Y	PARAMOUNT	Placebo (= BSC)
406	2016	Crizotinib	ALK	1st	1 Indication	PEM + PLAT	Y	PROFILE 1014	PEM + PLAT
411	2016	Necitumumab (+ gemcitabine + cisplatin for induction therapy, followed by necitumumab maintenance therapy)	Squamous with EGFR	1st	1 Indication	Gemcitabine + cisplatin	Y	SQUIRE	Placebo (+ gemcitabine + cisplatin for induction therapy, followed by maintenance therapy with necitumumab alone)
422	2016	Crizotinib	ALK	2nd	1 Indication	Docetaxel	N	PROFILE 1007	Docetaxel + PEM
428	2017	Pembrolizumab	PD-L1, with or without EGFR or ALK	2nd and later lines	Full population	Docetaxel	N	KEYNOTE-010 & KEYNOTE-011	Docetaxel
					Subgroup with adenocarcinoma histology	Nintedanib + docetaxel	N	KEYNOTE-010 & KEYNOTE-012	Docetaxel
500	2018	Ceritinib	ALK	1st	1 Indication	Crizotinib	N	ASCEND-4	Chemotherapy
520	2018	Atezolizumab	NSCLC	2nd	PD-L1 pos.	Pembrolizumab	N	OAK	Docetaxel
					PD-L1 pos.	Pembrolizumab	N	POPLAR	Docetaxel
					PD-L1 neg.	Docetaxel	Y	OAK	Docetaxel
					PD-L1 neg.	Docetaxel	Y	POPLAR	Docetaxel
531	2018	Pembrolizumab	PD-L1 ≥ 50%, no EGFR or ALK	1st	1 Indication	PEM + PLAT	Y	KEYNOTE-024	SOC: platinum-based combinations with either gemcitabine or paclitaxel, and PEM + PLAT (with/ without PEM maintenance for non-squamous disease).
529	2018	Crizotinib	ROS1	1st and later	Untreated	Chemotherapy	N	PROFILE 1001	Single arm trial
					Previously treated	Nintedanib + docetaxel	N	PROFILE 1014	PEM + PLAT - (in ALK population instead of ROS1 population)
					Previously treated	Docetaxel	N	PROFILE 1007	Docetaxel monotherapy, or nintedanib + docetaxel - (in ALK population instead of ROS1 population)
536	2018	Alectinib	ALK	1st	1 Indication	Crizotinib	Y	ALEX	Crizotinib
571	2019	Brigatinib	ALK	2nd	1 Indication	Ceritinib	N	ALTA	Single arm trial
					1 Indication	Ceritinib	N	Study-101	Single arm trial
578	2019	Durvalumab	PD-L1 ≥ 1%	2nd	1 Indication	BSC	Y	PACIFIC	BSC
584	2019	Atezolizumab (+ bevacizumab + carboplatin + paclitaxel)	PD-L1 (< 50%), EGFR, ALK	A. 1st when PD-L1 is 0- 49%; B. Later line when EGFR- or ALK-therapy failed.	1 Indication	PEM + PLAT, with/ without PEM maintenance	N	IMpower150	Bevacizumab + carboplatin + paclitaxel
595	2019	Dacominitib	EGFR	1st	1 Indication	Afatinib	N	ARCHER 1050	Gefitinib
					1 Indication	Erlotinib	N	ARCHER 1051	Gefitinib
					1 Indication	Gefitinib	Y	ARCHER 1052	Gefitinib
600	2019	Pembrolizumab (+ carboplatin + paclitaxel)	Squamous	1st	Squamous NSCLC with PD-L1	Platinum-based chemotherapy (carboplatin + gemcitabine, or carboplatin + vinorelbine)	N	KEYNOTE-407	Carboplatin + paclitaxel/nab-paclitaxel
					Squamous NSCLC with PD-L1 TPS 50–100%, no EGFR or ALK	Pembrolizumab	N	KEYNOTE-042	Pembrolizumab monotherapy versus standard chemotherapy (study needed to be: pembrolizumab combination versus pembrolizumab monotherapy)
628	2020	Lorlatinib	ALK	2nd and later	1 Indication	PDC	N	Study 1001	Single arm trial
					1 Indication	ABCP	N	Study 1001	Single arm trial
643	2020	Entrectinib	ROS1	1st	1 Indication	PEM + PLAT	N	STARTRK-2	Single arm trial
653	2020	Osimertinib	EGFR T790M	2nd	1 Indication	PDC	Y	AURA3	PDC
654	2020	Osimertinib	EGFR	1st	1 Indication	Gefitinib	Y	FLAURA	Gefitinib, or erlotinib
655	2020	Nivolumab	Squamous	2nd	1 Indication	Docetaxel	Y	CheckMate 017	Docetaxel
670	2021	Brigatinib	ALK	1st	1 Indication	Alectinib	N	ALTA-1 L	Crizotinib
					1 Indication	Crizotinib	Y	ALTA-1 L	Crizotinib
683	2021	Pembrolizumab (+ pemetrexed + platinum chemotherapy)	Non-squamous, no EGFR or ALK	1st	PDL1 neg.	PEM + PLAT, with/ without PEM maintenance	Y	KEYNOTE-189	PEM + PLAT
					PDL1 pos. with TPS ≤ 50%	PEM + PLAT, with/ without PEM maintenance	Y	KEYNOTE-189	PEM + PLAT
					PDL1 pos. with TPS ≥ 50%	Pembrolizumab	N	KEYNOTE-190	PEM + PLAT
705	2021	Atezolizumab	PD-L1 ≥ 50% or 10% of tumour-infiltrating immune cells, no EGFR or ALK	1st	1 Indication	Pembrolizumab	N	IMpower110	Chemotherapy
					1 Indication	Pembrolizumab + chemotherapy	N	IMpower110	Chemotherapy
713	2021	Nivolumab	Non-squamous PD-L1 positive	2nd	1 Indication	Docetaxel	Y	Checkmate 057	Docetaxel
724	2021	Nivolumab (+ ipilimumab + PDC)	NSCLC, no EGFR or ALK	1st	Non-squamous NSCLC with PDL1 TPS < 50%	PDC, with/ without PEM maintenance	Y	Checkmate-9LA	Standard PDC * Non-squamous NSCLC: PDC (PEM + PLAT, with optional PEM maintenance therapy. * Squamous NSCLC: PDC (paclitaxel + carboplatin)
					Non-squamous NSCLC with PDL1 TPS < 50%	ABCP	N	Checkmate-9LA	Standard PDC
					Non-squamous NSCLC with PDL1 TPS ≥ 50%	PDC, with/ without PEM maintenance	Y	Checkmate-9LA	Standard PDC
					Non-squamous NSCLC with PDL1 TPS ≥ 50%	Pembrolizumab	N	Checkmate-9LA	Standard PDC
					Non-squamous NSCLC with PDL1 TPS ≥ 50%	Pembrolizumab + PEM + PLAT	N	Checkmate-9LA	Standard PDC
					Squamous NSCLC with PDL1 TPS < 50%	PDC	Y	Checkmate-9LA	Standard PDC
					Squamous NSCLC with PDL1 TPS ≥ 50%	PDC	Y	Checkmate-9LA	Standard PDC
					Squamous NSCLC with PDL1 TPS ≥ 50%	Pembrolizumab	N	Checkmate-9LA	Standard PDC

Comments from the ERG (Evidence Review Group) and the NICE committee stated that the MAIC results were uncertain but in some cases were the only way to be able to compare treatments. Often the ERG concluded there is considerable uncertainty with performing indirect comparisons, and the outcomes should be viewed with caution. At the same time these indirect comparisons did support decision making and sometimes the committee concluded that this was the only way to be able to compare.

For twelve out of the 30 appraisals, NICE included two or more comparators. This concerned six appraisals for PD-(L)1 inhibitors (TA428, TA520, TA600, TA683, TA705 and TA724), and appraisals of treatments targeting ALK (TA628 and TA670), EGFR (TA595), ROS1 (TA529), and indications adenocarcinoma (TA347) and NSCLC (TA403). When focusing on inclusion of two or more comparators for PD-(L)1 inhibitors, this was often related to both the TPS (PD-L1 expression of ≥ 1%; 0–49%; or ≥ 50%) and squamous or non-squamous NSCLC status. For example, in TA600 pembrolizumab combined with carboplatin and paclitaxel was appraised for the indication ‘untreated metastatic squamous NSCLC’. To be able to include the preferred comparators, NICE needed to make a distinction in this population between PD-L1 TPS of 0–49%, and 50–100% with no EGFR or ALK mutations. The comparator considered most adequate for the PD-L1 TPS 0–49% population was platinum-based chemotherapy (carboplatin with gemcitabine, or carboplatin with vinorelbine), whereas the correct comparator for the PD-L1 TPS 50–100% concerned pembrolizumab monotherapy. The KEYNOTE-407 clinical trial that was performed to analyse the efficacy for this indication included placebo plus carboplatin and paclitaxel or nab-paclitaxel [46]. The KEYNOTE-042 trial was performed to analyse the efficacy of pembrolizumab monotherapy compared to standard chemotherapy. In this technology appraisal, two ITCs were performed, the first ITC to compare pembrolizumab combination therapy versus four chemotherapy comparators (1. gemcitabine plus cisplatin/carboplatin, 2. paclitaxel/nab-paclitaxel plus cisplatin/carboplatin, 3. docetaxel plus cisplatin/carboplatin, 4. vinorelbine plus cisplatin/carboplatin), whereas the second ITC compared pembrolizumab combination therapy versus pembrolizumab monotherapy. On top of that - separate NMAs were undertaken for: (a) patients with unselected histology and unselected PD-L1 status and (b) squamous histology and unselected PD-L1. Also, separate analyses were presented for progression-free survival (PFS) and overall survival (OS).

In five technology appraisals, the clinical evidence was coming from single arm trials. This concerned three appraisals for second line treatment of ALK positive NSCLC (TA395 in 2016, TA571 in 2019, and TA628 in 2020) and two appraisals for first line treatment of ROS1 positive NSCLC (TA529 in 2018 and TA643 in 2020). Both ITCs and MAICs were performed to be able to compare with the requested comparator in scope by NICE. The exception concerned TA529 of crizotinib for first line treatment of ROS1 positive NSCLC. In this appraisal the clinical trials of crizotinib for the patient population with ALK positive NSCLC were used as a proxy. The committee agreed that using data from a proxy population was not ideal but agreed to explore the proxy data in its decision-making, having considered the relatively small patient population. The committee stated that this should not set a precedent for the use of data from proxy populations in future appraisals.

In the other six appraisals that did not perform indirect comparisons, it was not deemed necessary to perform an indirect comparison as the comparator was included in the clinical trial in scope.

The use of indirect comparisons was common over the last decade. Over time, these indirect comparisons became more established in the form of NMAs and MAICs compared to the simpler comparisons that were conducted in the earlier years of the last decade.

### Patient reported outcome measures

All technology appraisals needed to include patient reported outcomes in the form of EuroQol-5 Dimension-3L (EQ-5D-3L) in the cost-effectiveness model. It appeared that use of EQ-5D in the economic models was predominantly in line with the NICE guidelines. Often other patient reported outcome measures (PROMs) were also considered in the technology appraisal to be able to further assess on lung cancer disease in specific, although these outcomes were mostly not included in the cost-effectiveness model. After EQ-5D, the PROMs EORTC core quality of life questionnaires C-30 (EORTC QLQ-C30) and LC-13 (EORTC QLQ-LC13) were most frequently included in clinical trials since 2015. Other PROMs that were used several times concerned the Lung Cancer symptom scale (LCSS) and the Functional Assessment of Cancer Therapy-Lung (FACT-L).

## Discussion

Over the last decade, 30 technology appraisals were performed for innovative NSCLC treatments in the UK. All these appraisals concerned NSCLC in the advanced and/or metastatic setting. Overall, 80% of appraisals were recommended for NHS, in addition three appraisals were recommended for use in CDF, and three appraisals were not recommended. Observed trends included first time appraisals for new mutations treated with targeted therapies concerned first line indications and for immunotherapy concerned second line; PartSA models were most frequently used and included most commonly three health transitions concerning PF, PD and death over a lifetime horizon with a cycle length of 7 days for immunotherapy and 28 to 30 days for targeted treatments; a total of 53 different comparators were included by NICE and 22 out of the 30 appraisals included indirect comparisons as not all comparators were investigated in head-to-head trials and for five appraisals the clinical evidence was coming from single arm trials; all appraisals included EQ-5D outcomes in the cost-effectiveness model and other patient reported outcomes that were considered most frequently concerned EORTC QLQ-C30 and LC-13.

Even though all new cancer drugs are considered for recommendation into the CDF, in practice not all new treatments were recommended for CDF uptake as 80% of appraisals were directly recommended for NHS. Recommendation for CDF was only the case when there was significant remaining clinical uncertainty which indirectly could lead to uncertainty around the end-of-life criteria and the cost-effectiveness.

Over these ten years, various methods to evaluate the cost-effectiveness of treatments were reported. With regards to cost-effectiveness models, the NICE Decision Support Unit (DSU) published a Technical Support Document (TSD 14) in 2011 in which it stated the preference of using PartSA modelling for cancer treatments [47]. It took until 2019 before most of the appraised cost-effectiveness models were in line with this recommendation in the field of NSCLC. However, PartSA models with the health transitions progression free, progressed disease and death became the cornerstone of health economic modelling in NSCLC. For the appraisals of PD-(L)1-inhibitors a possible new trend was seen with the implementation of the proximity of death approach. As not all technology appraisals for PD-(L)1 inhibitors used these time categories, it is not certain whether this will become a new trend within the appraisals of immunotherapies for NSCLC. Increasingly, treatment models for ALK positive NSCLC were more specifically designed for this individual mutation with applying a four health states model instead of three health states. Which is in line with the clinical trial endpoints of non-CNS progression and CNS progression. This could be an important new trend for the upcoming models for the treatment of NSCLC with ALK mutations, as CNS progression is a major cause of illness and death in this mutation specifically [48, 49]. Even though the perspective was mostly life long, we notified longer time horizons with up to 30 years for first line therapy and up to 20 years for second line, which is in line with the trend of improved survival in clinical trials. Cycle lengths differed from seven days for PD-(L)1 inhibitors, in line with administration every three or six weeks intravenously, to 28 or 30 days for inhibitors of the mutations EGFR, ALK or ROS1, which are administered orally daily. Our assumption was confirmed that when treatments for new mutations become available, NICE will first consider a WTP threshold of £50,000 per QALY. Our review showed that when more treatments for the same mutation will come to market, the WTP threshold for first line treatment decreased to £20,000–30,000 per QALY and for second line treatment was £50,000 per QALY as these were meeting the end-of-life criteria.

It was difficult to analyse ICER results as these were often only partially public and it was frequently unclear whether the presented ICER outcomes included a confidential discount. Therefore, the ICER results would be informative even though it would often not be the definite ICER for the intervention at scope.

Indirect comparisons were often conducted in the technology appraisals for NSCLC as direct head-to-head trials were often lacking for the various comparators or the subgroups that needed to be included for the appraisal. Although we expected that over time indirect comparisons would become more frequently applied due to the lack of active comparators, these comparisons were already performed in the earlier years of the last decade. Over time ITCs such as NMAs and MAICs became more established in comparison to the mixed treatment comparison and to more simple indirect comparisons. Especially since the first technology appraisals for immunotherapies in 2015 the treatment comparisons were getting more and more established. This complexity was due to the different indications and the various patient populations included in a technology appraisal, and the fast pace of updates in standard of care within NSCLC. This is in line with other disease areas within oncology where in a short period of time new treatments emerged that became the standard of care, for example in HER2-positive metastatic breast cancer and melanoma. [50–53]

Use of EQ5D in the economic models was predominantly in line with the NICE guidelines, and PROMs more focused on the quality of life in oncology and lung oncology were often captured by QLQ-C30 and QLQ-LC13 from 2015 onwards.

### Strengths and limitations

One of the strengths of this review is that we captured the finalised technology appraisals in the NICE database with a timespan of one decade (January 1st, 2012 - January 1st, 2022), enabling us to investigate the up-to-date trends of NSCLC treatments in the UK. However, we also have some limitations in this review. Firstly, our analysis only focused on the technology appraisals of NICE and therefore appraisals in journals and in other countries were not considered. Secondly, a challenge of this analysis was that the NICE database contains multiple documents and sometimes multiple review rounds were included, or two rounds of technology appraisals were necessary to reach the final appraisal determination. In these documents various perspectives and elaborations on certain topics were included from the Committee, the ERG, and the company. This made it very challenging to capture the proper information for our data extraction and in our analysis. Data from each technology appraisal was extracted from the most recent set of relevant papers from NICE. When specific data was not found in the most recent papers due to redacted text and data (black boxes) or references to earlier appraisal rounds, additional data was extracted from former available committee papers. Sometimes information could not be found in all the appraisal rounds due to confidentiality and redacted text. This limits our analyses of specific data or trends, but - we feel - not invalidates our general findings.

### Recommendations for future studies, policy, and practice

The technology appraisals that were needed to make decisions were both challenging for companies, ERG and NICE. This field is getting even more complex as new mutations and expressions are still being identified and innovative therapies are emerging within the field of NSCLC, such as treatments for the mutations or expressions BRAF, HER2, HER3, KRAS, MET, NTRK, RET, and TROP2. Some treatments are also pan-tumour molecular targets. Also, the appraisals over the last ten years included NSCLC in the advanced and/or metastatic setting. Currently, treatments are showing efficacy in the adjuvant and neo-adjuvant treatment setting, which will result in new treatment possibilities and therefore appraisals beyond the scope of advanced and/or metastatic NSCLC. It will be interesting to analyse future appraisals of advanced and/or metastatic NSCLC treatments considering these treatment options in earlier stages of NSCLC, together with the effect of lung cancer screening roll out leading to earlier diagnosis and improved outcomes in the UK [54]. 

Hence, in the years to come, a lot of new technology appraisals need to be performed not only just for the NSCLC disease area but also for overarching disease areas. It is not feasible to have one or two clinical trials for just one targeted treatment in NSCLC that will capture all the different patient (sub)populations or the different comparator treatments needed per subgroup, or to capture the current standard of care in such a rapidly changing disease area. Also, what is an acceptable way of appraising drugs for these oncogenes that will first have to be evaluated in single arm trials as there is currently no comparator? This leads to obstacles in the appraisal process with regards to clinical and scientific robustness and the timely matter in which these appraisals need to be performed. Therefore, the importance of indirect comparisons will increase even more. Our recommendation is to create more guidance for the elaboration of indirect comparisons on top of the existing guidance, like NICE DSU TSD 2 and 18, in order to reach more standardisation and transparency between appraisals, and that it is known which approaches and methods are acceptable [55, 56]. 

It can be questioned whether NICE needs a new approach with regards to appraising the emerging pan-tumour indications. NICE already recommended larotrectinib for use on the CDF for a range of cancers with the NTRK gene fusion mutation in May 2020 [57]. Other pan-tumour treatments are still being appraised per indication. One of the challenges that will also arise, is the need for including a recommended pan-tumour treatment as comparator in an appraisal for a targeted therapy in NSCLC. Further research needs to be performed to identify the obstacles and opportunities around this.

We have found that the patient reported outcomes used in the models were in line with the requirements of NICE. A deeper analysis into the use of PROMs is needed to further identify the trends and whether the impact of treatments on the quality of life of NSCLC patients can be captured in general with the required EQ5D utility values.

As already mentioned in this analysis, technology appraisals became more complicated. Further research is important to identify in what way the technology appraisal process can be simplified and be made more transparent and reproducible. NICE announced in January 2022 that it has rolled out changes to simplify and streamline processes to allow for more flexible decision making. Given the findings of this analysis this seems a great step in the right direction in this dynamic and challenging playing field [58]. 

## Conclusion

This study showed that over the last decade technology appraisals in the field of NSCLC became more complex due to the emergence of targeted therapies and immunotherapies that led to multiple different indications, subpopulations and comparators that needed to be included in appraisals, and the fast pace of updates in standard of care within NSCLC. Within the appraisals, PartSA models with three health states (PF, PD, Death) became the cornerstone within NSCLC, with time horizons up to 30 years and frequent use of NMAs and MAICs for indirect comparisons. Finally, with 90% positive recommendations for uptake either in NHS or CDF, the majority of treatments was recommended.

## Electronic supplementary material

Below is the link to the electronic supplementary material.

Supplementary Material 1

## Data Availability

All data used in this analysis were retrieved from public sources. Our data collection is available upon request.
