# Supplementary material for: Trends in NICE technology appraisals of non-small cell lung cancer drugs over the last decade
Source: Eur J Health Econ. 2024 Aug 30;26(3):455–71. doi: 10.1007/s10198-024-01711-0 (PMC11937191; doi:10.1007/s10198-024-01711-0)
Supplement: Supplementary file 1 — Supplementary Material 1 [file 10198_2024_1711_MOESM1_ESM.pdf]

# - Supplementary Material -

Trends in NICE technology appraisals of non-small cell lung cancer drugs over the last decade

Journal: European Journal of Health Economics

Authors: Westerink L, Wolters S, Zhou G, Postma AJ, Prof. dr. Boersma C, dr. van Boven JFM, Prof. dr. Postma MJ

Corresponding author: Lotte Westerink[1,2], l.westerink01@umcg.nl

ORCID: 0000-0001-9515-1044

1. Department of Health Sciences, University of Groningen, University Medical Center Groningen, Groningen, The Netherlands

2. AstraZeneca, Cambridge, United Kingdom.

Funding: This study was not funded.

Conflicts of interest: LW is currently employed at AstraZeneca, this research has been performed unrelated to her work at AstraZeneca and is part of her PhD research at the University Medical Center Groningen, the Netherlands. LW is a previous employee (at time of research) of Asc Academics Inc. SW and AP are employees of Asc Academics BV. GZ has no conflict of interest. MP received grants and honoraria from various pharmaceutical companies, including companies interested in the subject of this paper. JB received consultancy fees, honorarium, and research funding from Aardex, AstraZeneca, Chiesi, GSK, Novartis, Pfizer, Pill Connect, Teva, Trudell Medical and Vertex to consult, give lectures, provide

advice, and conduct independent research, all paid to his institution. CB received grants and honoraria from various pharmaceutical companies, including companies interested in the subject of this paper.

Trends in NICE technology appraisals of

non-small cell lung cancer drugs over the last decade

- Supplementary material -

Table of Contents

Appendix A – Section headings for data extraction 4
Appendix B – ICER outcomes 7
Appendix C – Indirect treatment comparisons 12

Appendix A – Section headings for data extraction

Table 1: Extraction form section headings

Overall information per drug per technology appraisal
• Generic name (+combination therapy)<br>• Generic name<br>• Monotherapy/ combination therapy<br>• Brand name<br>• Company<br>• Compound<br>• Line of treatment<br>• Mutation or expression
Information and recommendation NICE
• Number of Technology appraisal<br>• Data source<br>• Publication year<br>• Publication date<br>• Recommendation (+/-)<br>• Full recommendation stated<br>• Commercial arrangement<br>• Cancer Drug fund
Overview economic model
• Model approach<br>• Model health states & model transitions

• Time horizon<br>• Lifetime mentioned (Y/N)<br>• Cycle length (days)<br>• ICER/QALY (GBP)<br>• Willingness-to-pay threshold<br>• End-of-life criteria (Y/N)<br>• Details end-of-life criteria
Comparators and indirect comparisons for technology appraisal
• NICE comparators detailed<br>• NICE per row – sub indication<br>• NICE comparators per row<br>• Same as comparator in RCT (Y/N)<br>• RCT name<br>• RCT comparator<br>• Company's methods used for indirect comparison<br>• ERG/NICE on methods indirect comparison<br>• Indirect comparisons performed? (Y/N)<br>• Details indirect comparisons<br>• Feedback NICE on performed indirect comparisons
Patient reported outcomes for technology appraisal
• PRO detailed<br>• Utilities<br>• EQ5D-5L

- EQ5D-3L
- EORTC QLQ-C30
- EORTC QLQ-LC13
- Other QoL outcomes

Trends in NICE technology appraisals of

non-small cell lung cancer drugs over the last decade

- Supplementary material -

Appendix B – ICER outcomes

Table 1: Chronological overview of cost-effectiveness results in £ per QALY gained, ICERs expressed before confidential discount or with confidential discount included, depending on availability and transparency in technology appraisal.

Abbreviations: ABCP: atezolizumab + bevacizumab + carboplatin + paclitaxel, ALK: Anaplastic lymphoma kinase, BSC: Best supportive care, ECOG: Eastern Cooperative Oncology Group, EGFR: Epidermal growth factor receptor, Incl.: Including, NSCLC: Non-small cell lung cancer, Neg.: Negative, PAS: Patient access scheme, PD-L1: Programmed death-ligand 1, PDC: platinum-doublet chemotherapy, PEM: Pemetrexed, PLAT: Platinum chemotherapy, Pos.: Positive, QALY: Quality-adjusted Life Year, Ref.: Reference, ROS1: C-ros oncogene 1, TA: Technology appraisal, TKI: Tyrosine Kinase inhibitors, TPS: tumour proportion score, Vs.: versus (compared with), WTP: Willingness-to-pay.

TA	Publication year	Generic name (+combination therapy)	Indication specification	NICE comparators	ICER (£ per QALY gained)	WTP-threshold (£ per QALY gained)	Recommen-dation (+ / -)	Ref.
258	2012	Erlotinib	Locally advanced or metastatic EGFR-pos. NSCLC	Gefitinib	* £48,961 per QALY (no PAS included)<br>* £21,874 (incl. PAS)	20,000 - 30,000	+	(1)
310	2014	Afatinib	Locally advanced or metastatic EGFR-pos. NSCLC that did not receive EGFR TKIs previously	* Gefitinib, or erlotinib<br>* PEM + PLAT	* Vs. erlotinib: £10,076 per QALY<br>* Vs. gefitinib: £17,933 per QALY<br>* Vs. PEM + PLAT: £39,300 per QALY	20,000 - 30,000	+	(2)
347	2015	Nintedanib (+ docetaxel)	Locally advanced, metastatic, or recurrent NSCLC of adenocarcinoma histology that has porgressed after first-line chemotherapy	Docetaxel	£56,804 per QALY	50,000	+	(3)
395	2016	Ceritinib	Advanced ALK-pos. NSCLC who have previously had crizotinib	BSC	£86,400 per QALY	50,000	+	(4)
402	2016	PEM maintenance treatment	Maintenance treatment of locally advanced or metastatic non-squamous NSCLC in adults when: their disease has not progressed immediately after 4 cycles of pemetrexed and cisplatin induction therapy, their ECOG performance	BSC	£74,500 per QALY	50,000	+	(5)

			status is 0 or 1 at start of maintenance treatment					
403	2016	Ramucirumab (+ docetaxel)	Locally advanced or metastatic NSCLC progressing after platinum-based chemotherapy	* Docetaxel: full population, and subgroups with squamous & non-squamous NSCLC<br>* Nintedanib + docetaxel: subgroup with non-squamous NSCLC	* Vs. docetaxel for full population: £177,000 per QALY<br>* Vs. docetaxel subgroup non-squamous NSCLC: £148,000 per QALY<br>* Vs. nintedanib + docetaxel: £1,600,000 per QALY	*NSCLC or squamous NSCLC vs. docetaxel: 20,000 - 30,000<br>* Non-squamous NSCLC vs. nintedanib + docetaxel: 20,000 - 30,000<br>* Non-squamous NSCLC vs. docetaxel: 50,000	-	(6)
406	2017	Crizotinib	Untreated ALK-pos. NSCLC	PEM + PLAT	£47,291 per QALY	50,000	+	(7)
411	2016	Necitumumab (+ gemcitabine + cisplatin for induction therapy, followed by necitumumab maintenance therapy)	Locally advanced or metastatic EGFR-pos. squamous NSCLC that has not been treated with chemotherapy	Gemcitabine + cisplatin	Between £110,000 and £170,000 per QALY	20,000 - 30,000	-	(8)
422	2016	Crizotinib	Previously treated ALK-pos. NSCLC	* Docetaxel<br>* BSC	* Vs. docetaxel: > £100,000 per QALY<br>* Vs. BSC: > £50,200 per QALY	50,000	+	(9)
428	2017	Pembrolizumab	Locally advanced or metastatic PD-L1pos NSCLC after ≥1 chemotherapy (& targeted treatment if EGFR- or ALK-pos. tumour)	Docetaxel	Ranged from £44,490 per QALY for a three-year time horizon to £61,954 per QALY for a lifetime.	50,000	+	(10)
500	2018	Ceritinib	Untreated ALK-pos. NSCLC	Crizotinib	* With Gompertz extrapolation for OS: £58,808 per QALY<br>* With exponential extrapolation for OS: £37,410 per QALY	20,000 - 30,000	+	(11)
520	2018	Atezolizumab	Locally advanced or metastatic NSCLC after chemotherapy (& targeted treatment if EGFR- or ALK-pos. tumour)	* PD-L1 pos: pembrolizumab<br>* PD-L1 neg: docetaxel	*Vs. docetaxel - Base case, no stopping rule, list price: £168,591 per QALY<br>*Vs. docetaxel - Base case, 2 year stopping rule, list price: £151,662 per QALY<br>* Vs. pembrolizumab: -	* PD-L1 pos: 20,000 - 30,000<br>* PD-L1 neg: 50,000	+	(12)

531	2018	Pembrolizumab	Untreated PD-L1-pos. metastatic NSCLC with PD-L1 expression ≥ 50% TPS and no EGFR- or ALK-pos. mutations	PEM + PLAT	£30,244 per QALY gained (incl. 2 year stopping rule)	50,000	+	(13)
529	2018	Crizotinib	Advanced ROS1-pos. NSCLC	* Untreated: PEM + PLAT<br>* Previously treated: docetaxel, or nintedanib + docetaxel	* Vs. PEM + PLAT in untreated disease: ≥ £50,000 per QALY (exact ICERs are commercial in confidence)<br>* Vs. docetaxel in previously treated disease: well above £50,000 per QALY (exact ICERs are commercial in confidence)	50,000	+ for CDF	(14)
536	2018	Alectinib	Untreated ALK-pos. NSCLC	Crizotinib	Between £20,000 and £30,000 per QALY (exact ICERs are commercial in confidence)	20,000 - 30,000	+	(15)
571	2019	Brigatinib	Advanced ALK-pos. NSCLC after crizotinib	Ceritinib	Above £50,000 per QALY (exact ICERs are commercial in confidence)	50,000	+	(16)
578	2019	Durvalumab	Locally advanced unresectable NSCLC with PD-L1 expression of ≥ 1% TPS and whose disease has not progressed after platinum-based chemoradiation	BSC	* With log-normal extrapolation for PFS: above £30,000 per QALY<br>* With the gamma extrapolation: below £30,000 per QALY	20,000 - 30,000	+ for CDF	(17)
584	2019	Atezolizumab (+ bevacizumab + carboplatin + paclitaxel)	Metastatic non-squamous NSCLC when untreated with PD-L1 expression 0-49% TPS, or when targeted therapy for EGFR- or ALK-pos. NSCLC failed.	PEM + PLAT, with/ without PEM maintenance	Below £50,000 per QALY	50,000	+	(18)
595	2019	Dacominitib	Untreated advanced or metastatic EGFR-pos. NSCLC	Afatinib, or erlotinib, or gefitinib	Within range of £20,000 to £30,000 per QALY	20,000 - 30,000	+	(19)
600	2019	Pembrolizumab (+ carboplatin + paclitaxel)	Untreated metastatic squamous NSCLC	* Squamous NSCLC PD-L1 TPS 0-49%: platinum-based chemotherapy (carboplatin + gemcitabine, or carboplatin + vinorelbine)<br>* Squamous NSCLC PD-L1 TPS 50-100%, no EGFR or ALK: platinum-based chemotherapy, or	* Vs. standard care - base case for overall population: £25,431 per QALY<br>* Vs. standard care - subgroup PD-L1 TPS <1%: £47,252 per QALY<br>* Vs. standard care - subgroup PD-L1 TPS 1-49%: £30,201 per QALY<br>* Vs. standard care - subgroup PD-L1 TPS ≥50%: £15,623 per QALY<br>* Vs. pembrolizumab: -	50,000	+ for CDF	(20)

				pembrolizumab monotherapy	(Incl. PAS price for pembrolizumab & comparator list price)			
628	2020	Lorlatinib	ALK-pos. NSCLC in adults whose disease has progressed after 1. alectinib or ceritinib as the first ALK TKI or 2. crizotinib and ≥ 1 other ALK TKI	* PDC<br>* ABCP	* Vs. PDC: lowest £46,662 per QALY - highest £69,809 per QALY<br>* Vs. ABCP: lowest £12,505 per QALY - highest £13,978 per QALY<br>(Incl. confidential PAS for lorlatinib & list prices for comparator and subsequent treatments)	50,000	+	(21)
643	2020	Entrectinib	ROS1-pos. NSCLC in adults who have not had ROS1 inhibitors	PEM + PLAT	Range of £37,910 to £42,572 per QALY	50,000	+	(22)
653	2020	Osimertinib	EGFR T790M mutation-pos. locally advanced or metastatic NSCLC when progressed after first-line treatment with an EGFR TKI	PDC	Between £41,799 and £49,649 per QALY (incl. company's commercial arrangement)	50,000	+	(23)
654	2020	Osimertinib	Untreated locally advanced or metastatic EGFR-pos. NSCLC	Gefitinib	Within range of £20,000 to £30,000 per QALY	20,000 - 30,000	+	(24)
655	2020	Nivolumab	Locally advanced or metastatic squamous NSCLC after chemotherapy, only if:<br>it is stopped at 2 years of uninterrupted treatment, or earlier if their disease progresses and when patients have not had a PD-(L)1 inhibitor before.	Docetaxel	Below £40,168 per QALY	50,000	+	(25)
670	2021	Brigatinib	ALK-pos. NSCLC that has not been previously treated with an ALK inhibitor	Crizotinib	Below £20,000-£30,000 per QALY range (incl. confidential discounts for both brigatinib and crizotinib)	20,000 - 30,000	+	(26)
683	2021	Pembrolizumab (+ PEM + PLAT)	Untreated, metastatic, non-squamous NSCLC with no EGFR- or ALK-pos. mutations, only if: it is stopped at 2 years of uninterrupted treatment, or earlier if the disease progresses	* PD-L1 neg, or PD-L1 pos with TPS ≤50%: PEM + PLAT, with/ without PEM maintenance therapy<br>* PD-L1 pos with TPS ≥ 50% : pembrolizumab	* Vs. PEM + PLAT: Below **£**50,000 per QALY<br>* Vs. pembrolizumab monotherapy: between £20,000 and £30,000 per QALY<br>(incl. confidential discounts)	* PD-L1 pos: 20,000 - 30,000<br>* PD-L1 neg: 50,000	+	(27)

705	2021	Atezolizumab	Untreated metastatic NSCLC when PD-L1 expression ≥ 50% of tumour cells or 10% of tumour-infiltrating immune cells, and their tumours do not have EGFR- or ALK-pos. mutations	* Pembrolizumab<br>* Pembrolizumab + chemotherapy	Below £20,000 to 30,000 per QALY (incl. confidential discount)	20,000 - 30,000	+	(28)
713	2021	Nivolumab	Locally advanced or metastatic non-squamous NSCLC after chemotherapy, only if: their tumours are PD-L1 positive, it is stopped at 2 years of uninterrupted treatment, or earlier if their disease progresses, and patients did not have a PD-(L)1 inhibitor before.	Docetaxel	* With spline 3-knot hazard curve: £44,169 per QALY<br>* Log-normal curve: £44,547 per QALY	50,000	+	(29)
724	2021	Nivolumab (+ ipilimumab + PDC)	Untreated metastatic non-small-cell lung cancer (NSCLC) in adults whose tumours have no epidermal growth factor receptor (EGFR) or anaplastic lymphoma kinase (ALK) mutations	* Non-squamous NSCLC, with PD-L1 TPS <50%: PDC incl. optional PEM maintenance, or ABCP, or pembrolizumab + PEM + PLAT<br>* Squamous NSCLC, with PD-L1 TPS <50%: PDC<br>* Non-squamous NSCLC with PD-L1 TPS ≥50%: PDC incl. optional PEM maintenance, or pembrolizumab, or pembrolizumab + PEM + PLAT<br>* Squamous NSCLC with PD-L1 TPS ≥50%: PDC, or pembrolizumab	* Vs. ABCP and vs. PDC, for non-squamous NSCLC and PD-L1 TPS <50%: > £30,000 per QALY<br>* Vs. PDC for squamous NSCLC and PD-L1 TPS <50%: > £50,000 per QALY<br>* Vs. pembrolizumab for NSCLC of either histology and PD-L1 TPS ≥50%: more costly and less effective (was dominated by pembrolizumab)<br>* VS. PDC for NSCLC of either histology and PD-L1 TPS ≥50%: > £30,000 per QALY	* Squamous NSCLC, with PD-L1 TPS <50%: 50,000<br>* Non-squamous NSCLC, with PD-L1 TPS <50%: 20,000 - 30,000<br>* NSCLC of either histology, with PD-L1 TPS ≥50%: 20,000 - 30,000	-	(30)

Appendix C – Indirect treatment comparisons

Table 1: Chronological overview of performed indirect treatment comparisons (ITC) in the technology appraisals. Methods used for ITC, always concerned company methods and if available also ERG methods included.

Abbreviations: ABCP: atezolizumab + bevacizumab + carboplatin + paclitaxel, ALK: Anaplastic lymphoma kinase, BSC: Best supportive care, CNS: Central Nervous System, Combi: combination, ECOG: Eastern Cooperative Oncology Group, EGFR: Epidermal growth factor receptor, FP: fractional polynomial, HR: Hazard Ratio, Incl.: Including, ITC: Indirect treatment comparison, MAIC: Matching adjusted indirect comparison, MTC: Mixed treatment comparison, NMA: network meta-analysis, NSCLC: Non-small cell lung cancer, Neg.: Negative, OS: Overall survival, PD-L1: Programmed death-ligand 1, PFS: Progression free survival, PDC: platinum-doublet chemotherapy, PEM: Pemetrexed, PH: proportional hazards, PLAT: Platinum chemotherapy, Pos.: Positive, ROS1: C-ros oncogene 1, SOC: Standard of care TA: Technology appraisal, TKI: Tyrosine Kinase inhibitors, TPS: tumour proportion score, Vs.: versus (compared with).

TA	Publication year	Generic name (+combination therapy)	Indication focused on mutation/ expression	Line of treatment	ITCs performed (Y/N)	Methods used for ITC	Feedback NICE Committee on ITC Methods
258	2012	Erlotinib	EGFR	1st	Y	ITC	Not convinced that an ITC could be used with the existing data, to support the assumption that erlotinib was more effective than gefitinib, given the heterogeneity of the populations included and the variations in prognostic factors within the populations.
310	2014	Afatinib	EGFR	1st	Y	MTC	The underlying methodology of the MTC was not sufficiently robust
347	2015	Nintedanib (+ docetaxel)	Adenocarcinoma	2nd	Y	MTC	Docetaxel alone was the only appropriate comparator to nintedanib + docetaxel, and that it would not need to consider any comparison of nintedanib + docetaxel with erlotinib.
395	2016	Ceritinib	ALK	2nd	Y	Naïve ITC	The results of the naive indirect comparison, and specifically the size of benefit, were uncertain because there was a high risk of bias from confounding.
402	2016	Pemetrexed maintenance treatment	Non-squamous	Maintenance treatment	N	NA	NA

403	2016	Ramucirumab (+ docetaxel)	NSCLC	2nd	Y	NMA	The NMA was acceptable.
406	2017	Crizotinib	ALK	1st	N	NA	NA
411	2016	Necitumumab (+ gemcitabine + cisplatin for induction therapy, followed by necitumumab maintenance therapy)	Squamous with EGFR	1st	Y	NMA (Bayesian)	Noted important limitations in the NMA, and the results of the NMA were uncertain and it was difficult to draw conclusions from this analysis.<br>The platinum-combination regimens commonly used for squamous NSCLC are similar in effectiveness, and therefore concluded that it was sufficient to compare with gemcitabine + cisplatin using the direct evidence from SQUIRE.
422	2016	Crizotinib	ALK	2nd	Y	MTC	The MTC results were subject to uncertainty given the significant heterogeneity in the included studies.The resulting HR for OS for crizotinib vs. BSC should be viewed with considerable caution and that as a result, the relative effect remained an area of substantial uncertainty.
428	2017	Pembrolizumab	PD-L1, with or without EGFR or ALK	2nd and later lines	Y	NMA (Bayesian)	The ITC was not robust, and the trial populations of KEYNOTE-010 and LUME-LUNG-01 were too different. Therefore it was not appropriate for decision-making regarding the effectiveness of pembrolizumab compared with nintedanib in the population with adenocarcinoma.
500	2018	Ceritinib	ALK	1st	Y	2 MAICs	Results of both MAICs were subject to a high risk of bias because no common comparator arm in the trials being compared. The committee was aware of the issues with the MAIC and that the MAIC method is inappropriate without a common comparator, but concluded that an ITC of individual trial arms was the only way to compare ceritinib and crizotinib.
520	2018	Atezolizumab	NSCLC	2nd	Y	FP ITCs	Agreed to use the company's second updated network in its decision-making, but noted the uncertainty associated with all the indirect analyses. It concluded that atezolizumab may be clinically equivalent to pembrolizumab, but uncertainty remains.

531	2018	Pembrolizumab	PD-L1 ≥50%, no EGFR or ALK	1st	Y	1 Unadjusted NMA and 1 adjusted NMA	* ERG: Appropriate for the company to conduct NMA to support the existing direct evidence comparing pembrolizumab with the comparators of interest. The company's unadjusted and adjusted treatment crossover results are very similar raising concerns over the accuracy of the results. ERG notes that the results of the NMA were not used to inform the company's cost effectiveness base case.<br>* Committee: not available.
529	2018	Crizotinib	ROS1	1st and later	N	NA	Committee about clinical need: The ROS1 oncogene is a recent discovery and both patients and clinicians would welcome a targeted therapy
536	2018	Alectinib	ALK	1st	N	NA	NA
571	2019	Brigatinib	ALK	2nd	Y	Unanchored ITCs: a naive ITC and a MAIC	Given the available trial data, the company's approach was appropriate.
578	2019	Durvalumab	PD-L1 ≥1%	2nd	N	NA	NA
584	2019	Atezolizumab (+ bevacizumab + carboplatin + paclitaxel)	PD-L1 (<50%), EGFR, ALK	A. 1st when PD-L1 is 0%- 49% or B. Later line when EGFR- or ALK-therapy failed.	Y	FP NMA	The company's approach was appropriate.
595	2019	Dacominitib	EGFR	1st	Y	* Company: FP NMA<br>* ERG: fixed-effects NMA	* Committee on company ITC: The results from the company's FP NMA are uncertain.<br>* Committee on ERG ITC: Any estimates were uncertain and based on the evidence available there was no statistically significant difference between dacomitinib and afatinib in terms of extending PFS & OS.
600	2019	Pembrolizumab (+ carboplatin + paclitaxel)	Squamous	1st	Y	ITCs & NMAs (multiple)<br>* ITC1: Pembrolizumab combi vs. chemotherapy comparators<br>* ITC2: Pembrolizumab combi vs. pembrolizumab monotherapy<br>* Separate NMAs: (a) patients with unselected histology and unselected PD-L1 status and (b) squamous	* ERG: Concerns relating to the company's NMAs, in particular, the absence of second-line immunotherapy from the trials of SC chemotherapy comparator regimens.<br>* Committee: not available.

						histology and unselected PD-L1.<br>* Separate analyses: for PFS & OS	
628	2020	Lorlatinib	ALK	2nd and later	Y	MAICs & unadjusted ITCs (6 different approaches)<br>In addition to the MAIC, 2 further approaches were used for the ITC (6 methods):<br>* HR estimated using a MAIC with EXP-2:3A (method 1) and EXP-3B:5 (method 2)<br>* HRs estimated using an unadjusted ITC with EXP-2:3A (method 3) and EXP-3B:5 (method 4)<br>* Direct estimation of PFS & OS by fitting parametric curves to chemotherapy data from the clinical studies (method 5) and the same parametric curves with a population adjustment because the populations in these clinical studies had fewer prior treatments than the EXP-3B:5 cohort (method 6).	Methods 3, 4 and 6 had been dismissed at the technical engagement stage, it preferred method 5, mainly because of concerns about whether the assumption of PH held for the duration of the model with methods 1 and 2. The proposed ITC methods and the results from method 5 were highly uncertain.
643	2020	Entrectinib	ROS1	1st	Y	* Company: MAIC<br>* ERG: MAIC	Agreed that both company and ERG approaches have limitations but both should be considered for decision making.
653	2020	Osimertinib	EGFR T790M	2nd	Y	Adjusted ITC	Although it was not possible to determine which scenarios gave the most accurate estimate, the company's preferred adjustment was a reasonable estimate of survival.
654	2020	Osimertinib	EGFR	1st	N	NA	NA
655	2020	Nivolumab	Squamous	2nd	N	NA	NA
670	2021	Brigatinib	ALK	1st	Y	1 Unanchored MAIC, 1 anchored MAIC, 1 unweighted Bucher ITC	The unanchored MAIC results were not acceptable for decision making. The committee accepted that, considering that brigatinib and alectinib have similar mechanisms of action, an increase in PFS and CNS PFS could plausibly translate to a benefit in OS, although uncertainty remains about this. It also accepted that it was

							plausible for similar OS to be seen with brigatinib and alectinib, given the similarities between the 2 treatments.
683	2021	Pembrolizumab (+ pemetrexed + platinum chemotherapy)	Non-squamous, no EGFR or ALK	1st	Y	ITC	The company's ITC showed no statistically significant difference in OS between pembrolizumab combi and pembrolizumab monotherapy in people with ≥ 50% PD-L1 TPS. Therefore, the additional 3-month survival gain for pembrolizumab combi therapy vs. pembrolizumab was uncertain.
705	2021	Atezolizumab	PD-L1 ≥50% or 10% of tumour-infiltrating immune cells, no EGFR or ALK	1st	Y	1 standard NMA & 1 FP NMA	This approach was considered to be acceptable for use in decision making. Overall, the committee agreed with the ERG and concluded that the results from the NMA suggested no significant differences between atezolizumab and pembrolizumab.
713	2021	Nivolumab	Non-squamous PD-L1 positive	2nd	N	NA	NA
724	2021	Nivolumab (+ ipilimumab + PDC)	NSCLC, no EGFR or ALK	1st	Y	2 ITCs: 1 vs. pembrolizumab monotherapy, 1 vs. atezolizumab combination	Some of the company's ITC results had wide confidence intervals and were uncertain, but concluded that they were acceptable for decision making. The ITC with pembrolizumab + PEM + PLAT is not suitable for decision making.

References

1. NICE. Erlotinib for the first-line treatment of locally advanced or metastatic EGFR-TK mutation-positive non-small-cell lung cancer; Technology appraisal guidance [TA258] [Internet]. nice.org.uk. 2012 [cited 2023 May 29]. Available from: https://www.nice.org.uk/guidance/ta258/history
2. NICE. Afatinib for treating epidermal growth factor receptor mutation-positive locally advanced or metastatic non-small-cell lung cancer; Technology appraisal guidance [TA310] [Internet]. nice.org.uk. 2014 [cited 2022 Jan 27]. Available from: https://www.nice.org.uk/guidance/ta310
3. NICE. Nintedanib for previously treated locally advanced, metastatic, or locally recurrent non-small-cell lung cancer; Technology appraisal guidance [TA347] [Internet]. nice.org.uk. 2015 [cited 2022 Jan 27]. Available from: https://www.nice.org.uk/guidance/ta347
4. NICE. Ceritinib for previously treated anaplastic lymphoma kinase positive non-small-cell lung cancer; Technology appraisal guidance [TA395] [Internet]. nice.org.uk. 2016 [cited 2022 Jan 27]. Available from: https://www.nice.org.uk/guidance/ta395
5. NICE. Pemetrexed maintenance treatment for non-squamous non-small-cell lung cancer after pemetrexed and cisplatin; Technology appraisal guidance [TA402] [Internet]. nice.org.uk. 2016 [cited 2022 Jan 27]. Available from: https://www.nice.org.uk/guidance/ta402
6. NICE. Ramucirumab for previously treated locally advanced or metastatic non-small-cell lung cancer; Technology appraisal guidance [TA403] [Internet]. nice.org.uk. 2016 [cited 2022 Jan 27]. Available from: https://www.nice.org.uk/guidance/ta403
7. NICE. Crizotinib for untreated anaplastic lymphoma kinase-positive advanced non-small-cell lung cancer; Technology appraisal guidance [TA406] [Internet]. nice.org.uk. 2016 [cited 2022 Jan 27]. Available from: https://www.nice.org.uk/guidance/ta406
8. NICE. Necitumumab for untreated advanced or metastatic squamous non-small-cell lung cancer; Technology appraisal guidance [TA411] [Internet]. nice.org.uk. 2016 [cited 2022 Jan 27]. Available from: https://www.nice.org.uk/guidance/ta411
9. NICE. Crizotinib for previously treated anaplastic lymphoma kinase-positive advanced non-small-cell lung cancer; Technology appraisal guidance [TA422] [Internet]. nice.org.uk. 2016 [cited 2022 Jan 27]. Available from: https://www.nice.org.uk/guidance/ta422
10. NICE. Pembrolizumab for treating PD-L1-positive non-small-cell lung cancer after chemotherapy; Technology appraisal guidance [TA428] [Internet]. nice.org.uk. 2017 [cited 2022 Jan 27]. Available from: https://www.nice.org.uk/guidance/ta428/history

11. NICE. Ceritinib for untreated ALK-positive non-small-cell lung cancer; Technology appraisal guidance [TA500] [Internet]. nice.org.uk. 2018 [cited 2022 Jan 27]. Available from: https://www.nice.org.uk/guidance/ta500

12. NICE. Atezolizumab for treating locally advanced or metastatic non-small-cell lung cancer after chemotherapy; Technology appraisal guidance [TA520] [Internet]. nice.org.uk. 2018 [cited 2022 Jan 27]. Available from: https://www.nice.org.uk/guidance/ta520

13. NICE. Pembrolizumab for untreated PD-L1-positive metastatic non-small-cell lung cancer; Technology appraisal guidance [TA531] [Internet]. nice.org.uk. 2018 [cited 2022 Jan 27]. Available from: https://www.nice.org.uk/guidance/ta531

14. NICE. Crizotinib for treating ROS1-positive advanced non-small-cell lung cancer; Technology appraisal guidance [TA529] [Internet]. nice.org.uk. 2018 [cited 2022 Jan 27]. Available from: https://www.nice.org.uk/guidance/ta529

15. NICE. Alectinib for untreated ALK-positive advanced non-small-cell lung cancer; Technology appraisal guidance [TA536] [Internet]. nice.org.uk. 2018 [cited 2022 Jan 27]. Available from: https://www.nice.org.uk/guidance/ta536

16. NICE. Brigatinib for treating ALK-positive advanced non-small-cell lung cancer after crizotinib; Technology appraisal guidance [TA571] [Internet]. nice.org.uk. 2019 [cited 2022 Jan 27]. Available from: https://www.nice.org.uk/guidance/ta571

17. NICE. Durvalumab for treating locally advanced unresectable non-small-cell lung cancer after platinum-based chemoradiation; Technology appraisal guidance [TA578] [Internet]. nice.org.uk. 2019 [cited 2022 Jan 27]. Available from: https://www.nice.org.uk/guidance/ta578

18. NICE. Atezolizumab in combination for treating metastatic non-squamous non-small-cell lung cancer; Technology appraisal guidance [TA584] [Internet]. nice.org.uk. 2019 [cited 2022 Jan 27]. Available from: https://www.nice.org.uk/guidance/ta584

19. NICE. Dacomitinib for untreated EGFR mutation-positive non-small-cell lung cancer; Technology appraisal guidance [TA595] [Internet]. nice.org.uk. 2019 [cited 2022 Jan 27]. Available from: https://www.nice.org.uk/guidance/ta595

20. NICE. Pembrolizumab with carboplatin and paclitaxel for untreated metastatic squamous non-small-cell lung cancer; Technology appraisal guidance [TA600] [Internet]. nice.org.uk. 2019 [cited 2022 Jan 27]. Available from: https://www.nice.org.uk/guidance/ta600

21. NICE. Lorlatinib for previously treated ALK-positive advanced non-small-cell lung cancer; Technology appraisal guidance [TA628] [Internet]. nice.org.uk. 2020 [cited 2022 Jan 27]. Available from: https://www.nice.org.uk/guidance/ta628

22. NICE. Entrectinib for treating ROS1-positive advanced non-small-cell lung cancer; Technology appraisal guidance [TA643] [Internet]. nice.org.uk. 2020 [cited 2022 Jan 27]. Available from: https://www.nice.org.uk/guidance/ta643

23. NICE. Osimertinib for treating EGFR T790M mutation-positive advanced non-small-cell lung cancer; Technology appraisal guidance [TA653] [Internet]. nice.org.uk. 2020 [cited 2022 Jan 27]. Available from: https://www.nice.org.uk/guidance/ta653

24. NICE. Osimertinib for untreated EGFR mutation-positive non-small-cell lung cancer; Technology appraisal guidance [TA654]. nice.org.uk. 2020.

25. NICE. Nivolumab for advanced squamous non-small-cell lung cancer after chemotherapy; Technology appraisal guidance [TA655] [Internet]. nice.org.uk. 2020 [cited 2022 Jan 27]. Available from: https://www.nice.org.uk/guidance/ta655

26. NICE. Brigatinib for ALK-positive advanced non-small-cell lung cancer that has not been previously treated with an ALK inhibitor; Technology appraisal guidance [TA670] [Internet]. nice.org.uk. 2021 [cited 2022 Jan 27]. Available from: https://www.nice.org.uk/guidance/ta670

27. NICE. Pembrolizumab with pemetrexed and platinum chemotherapy for untreated, metastatic, non-squamous non-small-cell lung cancer; Technology appraisal guidance [TA683] [Internet]. nice.org.uk. 2021 [cited 2022 Jan 27]. Available from: https://www.nice.org.uk/guidance/ta683

28. NICE. Atezolizumab monotherapy for untreated advanced non-small-cell lung cancer; Technology appraisal guidance [TA705] [Internet]. nice.org.uk. 2021 [cited 2022 Jan 27]. Available from: https://www.nice.org.uk/guidance/ta705

29. NICE. Nivolumab for advanced non-squamous non-small-cell lung cancer after chemotherapy; Technology appraisal guidance [TA713] [Internet]. nice.org.uk. 2021 [cited 2022 Jan 27]. Available from: https://www.nice.org.uk/guidance/ta713

30. NICE. Nivolumab with ipilimumab and chemotherapy for untreated metastatic non-small-cell lung cancer; Technology appraisal guidance [TA724] [Internet]. nice.org.uk. 2021 [cited 2022 Jan 27]. Available from: https://www.nice.org.uk/guidance/ta724
